# New Sight: Enzymes as Targets for Drug Development

**DOI:** 10.3390/cimb45090482

**Published:** 2023-09-20

**Authors:** Sung-Kun Kim

**Affiliations:** Department of Natural Sciences, Northeastern State University, Broken Arrow, OK 74014, USA; kim03@nsuok.edu; Tel.: +1-918-449-6414

In the dynamic realm of medical research, a resounding chord is struck by recent studies that have propelled drug discovery to new horizons across a spectrum of disciplines. At the forefront of this progress lies a growing reliance on computational methodologies, which are illuminating the path to unravel intricate biological processes and accelerating the development of cutting-edge treatments. These studies seamlessly combine the power of computational techniques with traditional wet-lab experiments, offering a more streamlined and efficient approach to deciphering the complex interactions between molecules and enzymes within living systems.

Enzymes, the orchestrators of vital biochemical reactions within the intricate cellular milieu, operate not in isolation but within a broader context. The exploration of enzyme inhibition through cell-level investigations opens a window into the ripple effects of enzyme inhibitors within the dynamic landscape of the cell. These effects extend beyond the target enzyme, resonating across cellular components, pathways, and interactions. This holistic understanding is essential for comprehending both the potential advantages and limitations of enzyme inhibitors when employed in living systems. Moreover, the realm of enzyme kinetics, which delves into understanding reaction rates, becomes instrumental in characterizing the inhibitory effects of potential drugs. Through meticulous kinetic analyses, critical insights are gleaned regarding inhibitor binding, inhibition mechanisms, and overall efficiency, all of which are indispensable for the fine-tuning of inhibitor design.

In the quest to decipher the intricate world of enzymes and their modulation, computational techniques stand as formidable allies. Molecular docking and dynamic simulations take center stage, providing essential tools for enzyme characterization and drug development. These computational methods forecast interactions, elucidate binding mechanisms, and predict molecular stability, thus accelerating the identification of potential drug candidates. The precision gained from accurate enzyme characterization is imperative to grasp the nuances of enzyme structure, function, and interactions, which in turn inform the strategic design of effective inhibitors. Given the pivotal roles enzymes play in various diseases and biological processes, their precise characterization acts as a guiding beacon for compound design, ultimately facilitating therapeutic intervention.

The culmination of these groundbreaking endeavors is captured within the pages of this Special Issue, offering a treasure trove of knowledge that delves into the realm of enzyme inhibition through the lens of cell-level studies. These investigations bring to light a profound understanding of how enzyme inhibitors intricately weave their influence within the dynamic tapestry of living organisms. Enzymes, often likened to the molecular architects steering vital biochemical reactions, are under meticulous regulatory control to maintain physiological equilibrium. Inhibition, a pivotal mechanism of regulation, casts its influence across the diverse biological processes and pathways associated with diseases.

Among the many exemplars, Epimakhova et al. cast a brilliant spotlight on the interplay between oxidative stress and schizophrenia, unraveling catalytically active antibodies or abzymes that present intriguing redox properties within the serum [1]. This study dives deep into the core of this phenomenon, exploring the effects of these immunoglobulins on neuroblastoma cells. The intricate narrative of cytotoxicity emerges, entwined with the potent catalytic effects of superoxide dismutase (SOD). This study’s revelation of the divergent cytotoxic impacts of immunoglobulins from schizophrenia patients and healthy individuals under oxidative stress provides a fresh lens through which to understand the complex relationship between abzymes and cellular wellbeing and offers new perspectives into the mechanisms underpinning oxidative stress in schizophrenia.

Moreover, the complexity of enzyme inhibition extends further into the field of schizophrenia research with investigations into antibodies targeting myelin basic protein (MBP) [2]. This study unravels the cryptic behavior of these antibodies and their proteolytic tendencies towards MBP. By meticulously dissecting IgG peptides within blood serum, specific sequences linked to MBP hydrolysis are uncovered. These sequences, predominantly found within IgG heavy and light chains, bear a substantial association with MBP hydrolysis. While the peptide content of light chain variable regions shows no direct correlation with proteolytic activity, intriguingly, two sequences originating from heavy chain variable regions exhibit heightened activity at higher concentrations. These findings offer tantalizing insights into the intricate interplay that might unlock the enigma of MBP hydrolysis in schizophrenia, stimulating further exploration.

In the broader context of drug discovery, the spotlight is cast on the vital role played by enzyme kinetics in bridging the gap between enzymology and therapeutic intervention. In this Special Issue, the work of Kim et al. presents a captivating exploration of the inhibitory capabilities of quaternary isoquinoline alkaloids against Soluble Epoxide Hydrolase (sEH) [3]. Through meticulous analyses of enzyme kinetics, a noncompetitive inhibition pattern is unveiled, laying the groundwork for potential therapeutic strategies. Parallelly, in the realm of Soluble Epoxide Hydrolase (sEH) modulation, the study by Kim et al. presents a comprehensive review that intricately weaves the role of sEH across diverse disorders, spotlighting representative inhibitors and igniting future inhibitor development [4]. Notably, the study by Kim et al. sheds light on the therapeutic potential of sEH in the context of neuroinflammation and Alzheimer’s disease, offering a fresh perspective on its role and potential as a therapeutic target [5].

In the ever-evolving realm of medical research, a noteworthy theme takes center stage, spotlighting recent advancements that hold the potential to revolutionize drug discovery across diverse domains. At the core of this progress lies an emerging trend—the increasing reliance on computational methodologies. These techniques play a pivotal role in unraveling the intricate tapestry of biological processes, expediting the development of groundbreaking treatments. This symbiotic integration of computational methodologies with traditional wet-lab experiments ushers in a more streamlined and efficient approach to decoding the intricate web of molecular and enzymatic interactions.

A significant contribution in this arena comes from Deng et al., who embarked on an exploratory expedition into the complex domain of drug–target interactions—an indispensable aspect of drug development [6]. Acknowledging the limitations of conventional experimental approaches in meeting the demands of modern drug research, the researchers introduce a revolutionary solution—DeepMHADTA. This cutting-edge deep learning model incorporates multi-head self-attention within a deep residual network, promising the unprecedented ability to forecast drug–target binding affinity with unparalleled precision. Impressively, DeepMHADTA not only surpasses existing techniques on benchmark datasets but also demonstrates remarkable accuracy in predicting drug–target interactions.

Further highlighting the predictive prowess of computational methodologies is the arena of drug–target binding affinity prediction. As a pivotal element of drug repositioning and screening, prevailing methods often lean towards binary classification or regression for binding anticipation, with regression offering deeper insights. In response, a novel strategy called Affinity2Vec emerges. This approach, characterized by a regression-based framework utilizing a weighted heterogeneous graph, effectively anticipates binding affinity without necessitating exhaustive target structural information [7]. Affinity2Vec’s impressive performance on benchmark datasets underscores its efficacy and robustness, validated through multiple evaluation metrics.

Underscoring the significance of predicting drug–target interactions for drug discovery and repurposing, another study emphasizes the pivotal role played by binding affinity in gauging these interactions. Within this context, a novel approach named NerLTR-DTA takes the spotlight. Leveraging neighbor relationships and a ranking framework, this method accurately predicts both affinity values and the priority order of query drugs or targets alongside associated proteins or compounds [8]. Demonstrating superior performance across diverse scenarios, NerLTR-DTA emerges as a promising tool for facilitating new drug exploration and repurposing. By furnishing precise ranking lists based on association relationships, this methodology charts a promising trajectory within the intricate landscape of drug research.

Overall, in conclusion, this compilation of studies ushers us into the heart of enzyme inhibition, where cells become the canvas of intricate interactions. The dynamic interplay between inhibitors and enzymes within living systems offers a wealth of insights that shape the landscape of biomedical understanding. As we peer deeper into the molecular realm, we uncover new vistas of therapeutic potential, poised to revolutionize the fight against diseases that touch millions worldwide. In the grand tapestry of enzymology, these studies illuminate the pivotal role of enzyme kinetics and catalytic specificity. By unraveling the dynamic interactions between enzyme inhibitors and their targets, they offer a glimpse into the intricate choreography governing biochemical reactions. As the world of scientific discovery continues to evolve, these insights serve as beacons guiding researchers toward the development of innovative therapeutic interventions and novel treatments, poised to revolutionize our approach to complex health challenges. These recent studies exemplify the transformative impact of computational methodologies in advancing drug discovery. With a keen focus on drug–target interactions, antiviral strategies, cancer therapies, and diabetic complications, these investigations collectively contribute to a deeper understanding of intricate biological processes. As scientific research continues to evolve, these studies underscore the role of computational tools as vital companions to experimental approaches, paving the way for a more efficient and effective drug discovery landscape that holds the promise of improving global health outcomes.
